# Unraveling the mechanisms underlying drug-induced cholestatic liver injury: identifying key genes using machine learning techniques on human in vitro data sets

**DOI:** 10.1007/s00204-023-03583-4

**Published:** 2023-08-21

**Authors:** Jian Jiang, Jonas van Ertvelde, Gökhan Ertaylan, Ralf Peeters, Danyel Jennen, Theo M. de Kok, Mathieu Vinken

**Affiliations:** 1 Entity of In Vitro Toxicology and Dermato‑Cosmetology, Department of Pharmaceutical and Pharmacological Sciences, Vrije Universiteit Brussel, Laarbeeklaan 103, 1090 Brussels, Belgium; 2 Vlaamse Instelling voor Technologisch Onderzoek (VITO) NV, Health, Boeretang 200, 2400 Mol, Belgium; 3 Maastricht Centre for Systems Biology (MaCSBio), Maastricht University, Maastricht, The Netherlands; 4 Department of Advanced Computing Sciences, Maastricht University, Maastricht, The Netherlands; 5 Department of Toxicogenomics, GROW School for Oncology and Reproduction, Maastricht University, Maastricht, The Netherlands

**Keywords:** Drug-induced cholestasis, Feature selection, Machine learning, Supervised classification, Wrapper feature selection

## Abstract

Drug-induced intrahepatic cholestasis (DIC) is a main type of hepatic toxicity that is challenging to predict in early drug development stages. Preclinical animal studies often fail to detect DIC in humans. In vitro toxicogenomics assays using human liver cells have become a practical approach to predict human-relevant DIC. The present study was set up to identify transcriptomic signatures of DIC by applying machine learning algorithms to the Open TG-GATEs database. A total of nine DIC compounds and nine non-DIC compounds were selected, and supervised classification algorithms were applied to develop prediction models using differentially expressed features. Feature selection techniques identified 13 genes that achieved optimal prediction performance using logistic regression combined with a sequential backward selection method. The internal validation of the best-performing model showed accuracy of 0.958, sensitivity of 0.941, specificity of 0.978, and F1-score of 0.956. Applying the model to an external validation set resulted in an average prediction accuracy of 0.71. The identified genes were mechanistically linked to the adverse outcome pathway network of DIC, providing insights into cellular and molecular processes during response to chemical toxicity. Our findings provide valuable insights into toxicological responses and enhance the predictive accuracy of DIC prediction, thereby advancing the application of transcriptome profiling in designing new approach methodologies for hazard identification.

**Supplementary Information:**

The online version contains supplementary material available at 10.1007/s00204-023-03583-4.

## Introduction

Drug-induced liver injury (DILI) accounts for over 50% of all cases of acute liver failure cases in Western countries (Vinken 2018). This clinical concern causes one in three market withdrawals during pre-marketing and post-marketing phases, resulting in significant costs for pharmaceutical companies (Dirven et al. 2021). Depending on the pathological patterns of liver injury, DILI can be classified into three categories, namely cholestatic, hepatocellular, and a mixed type of injury (Kullak-Ublick 2013). Drug-induced intrahepatic cholestasis (DIC), characterized by bile acid (BA) accumulation in the liver, constitutes a major subgroup of total DILI cases (Gijbels et al. 2020). It occurs when a drug disturbs BA homeostasis, leading to an increase in hepatotoxic effects of BAs (Shin et al. 2020).

Currently, preclinical drug toxicity testing relies heavily on animal models (Dirven et al. 2021). Despite posing a serious ethical problem, these animal-based toxicity predictions have shown limited relevance for humans, likely due to the significant interspecies-related differences in hepatocellular function, drug metabolism, and pharmacokinetics (Perez Santin et al. 2021). Preclinical animal studies indeed often fail to detect DIC due to substantial variances in tissue-specific BA compositions and levels as well as in the subsequent cellular responses between these laboratory animals and humans (Thakare et al. 2018).

To address this issue, substantial efforts have been devoted to developing and implementing new approach methodologies aiming to move away from animal testing toward animal-free and human-relevant in vitro assays, in silico methods, and other biotechnological and computational approaches in chemical hazard assessment (Andersen et al. 2019). In vitro toxicogenomics, particularly those using human liver cells, have become a more convenient and practical approach to assess and predict human-relevant DILI. Transcriptomic analysis, providing information on global gene expression profiles in response to a compound exposure, has facilitated our molecular understanding of toxicological mechanisms and has shown potential in advancing drug safety assessment. For example, human in vitro transcriptomics-based tests have produced promising results in differentiating between genotoxic and non-genotoxic chemicals (Magkoufopoulou et al. 2012; Van den Hof et al. 2014). Despite these positive developments, implementing transcriptomics measurements in large-scale risk assessment workflows still is challenging. In this respect, conventional differential expression analysis usually leads to outcomes consisting of hundreds or even thousands of genes, making it unsuitable for high-throughput laboratory testing (Smith et al. 2020). Machine learning (ML), a branch of artificial intelligence, enables computers to learn from data and make predictions with minimal human intervention (Wu and Wang 2018). Applying ML approaches to transcriptomic profiling in toxicity studies allows recognizing distinct molecular patterns associated with drug-induced toxicity. Moreover, feature elimination algorithms, which are techniques enabling to identify key features that contribute to the disease of interest, can assist in reducing the feature size used in hazard prediction (Yang et al. 2015).

The goal of this study was to construct a classifier from high-dimensional microarray data to improve hepatotoxicity prediction and use feature elimination algorithms to identify key feature genes for DIC. In particular, we propose a hybrid approach leveraging (i) a wild list of DIC-associated genes identified by differential expression analysis, (ii) an optimal subset of differentially expressed genes (DEGs) with maximum relevance for predicting the target variable selected using supervised ML methods, and (iii) evaluation of the discriminatory power of the established model and the selected DIC signature. To this end, we mined the publicly available database Open Toxicogenomics Project-Genomics Assisted Toxicity Evaluation Systems (TG-GATEs) (Igarashi et al. 2015), which contains microarray-based gene expression profiles of primary human hepatocytes (PHHs) in response to over 150 chemical compounds. The genes of interest were benchmarked against previously introduced adverse outcome pathway(AOP) network for DIC (Gijbels et al. 2020; Vinken et al. 2013) that have indicated that a number of molecular initiating events (MIEs) (i.e., transporter changes, hepatocellular changes and bile canalicular alterations) and key events (KEs) (i.e., inflammation, mitochondrial impairment, oxidative stress, endoplasmic reticulum (ER) stress and the simultaneously triggered adaptive response) driving the pathogenesis of DIC.

**Fig. 1:**
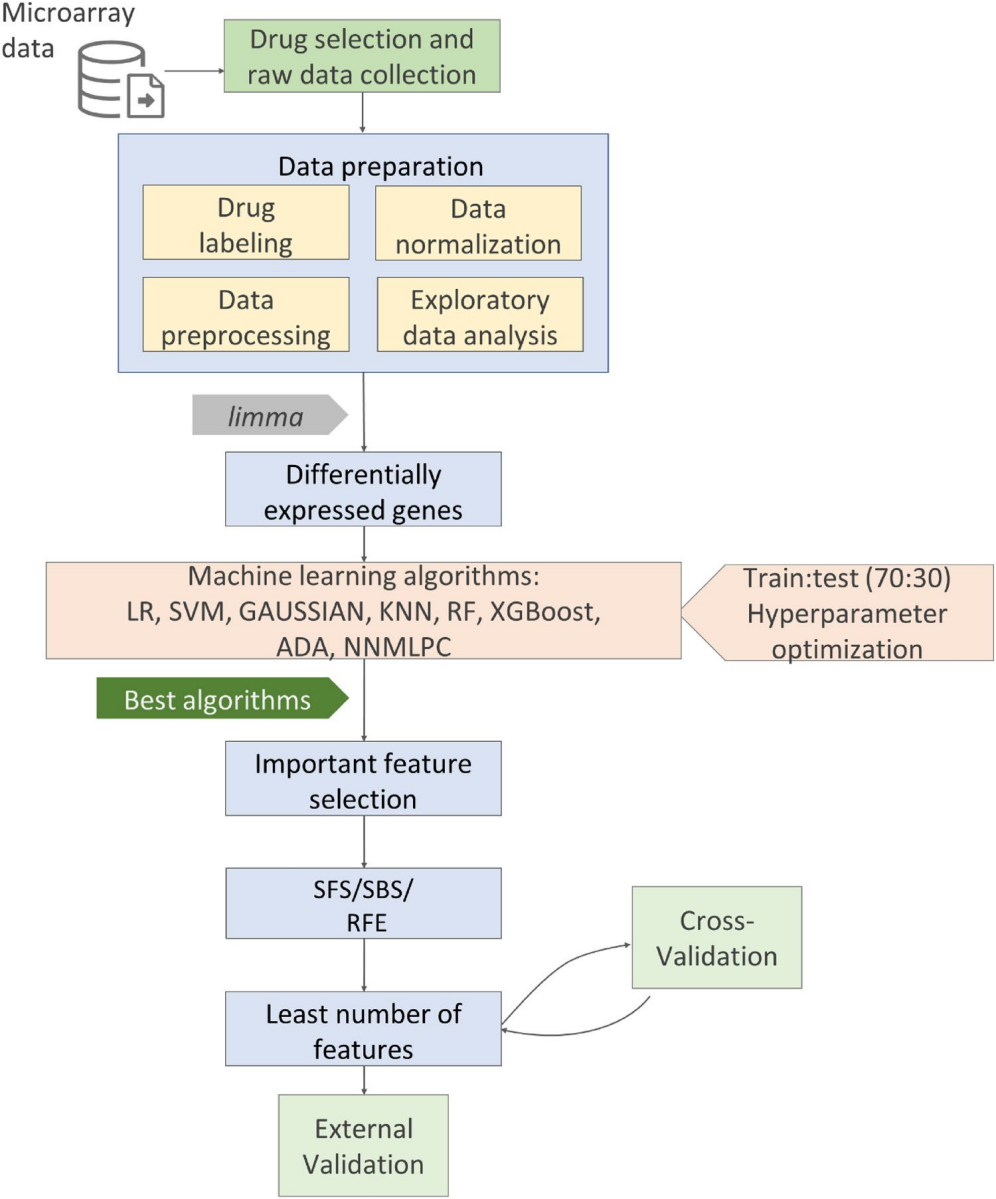
Workflow of this study. Various machine learning algorithms and feature selection methods were employed. *ADA* AdaBoost, *Gaussian* Gaussian process classifier, *GBC* gradient boosting classifier, *KNN* K-nearest neighbors, *LR* logistic regression, *NNMLPC* neural network multilayer perceptron classifier, *RF* random forests, *RFE* recursive feature elimination, *SBS* sequential backward selection, *SFS* sequential forward selection, *SVM* support vector machine, *XGBoost* extreme gradient boosting

The overall workflow of this study is presented in Fig. 1.

## Materials and methods

### Compound selection and labeling

A total of 18 compounds, consisting of 9 DIC and 9 non-DIC compounds, were utilized to create the training set, as they had transcriptome data accessible in the Open TG-GATEs database. To assess the model’s generalizability, gene expression profiles from human hepatoma HepG2 cells treated with ten compounds (five DIC and five non-DIC) were retrieved from the GEO database to form an external test set.

Compounds in the training set were labeled DIC-positive when their cholestasis mechanisms in humans were relatively clear. DIC-negative drugs met either of two criteria: (i) classified as ‘No-DILI concern’ in DILIrank, a database categorizing the DILI potential of over 1000 FDA-approved drugs (Chen et al. 2016), or (ii) no hepatotoxicity and genotoxicity reports were found in the LiverTox database (Hoofnagle et al. 2013) and EURL ECVAM Ames-positives consolidated genotoxicity and carcinogenicity database (Madia et al. 2020).

To map the full mechanistic scenario of DIC, nine model cholestatic compounds were chosen for their known abilities to induce DIC in primary human hepatocytes through diverse toxic mechanisms. The positive compounds include (i) chlorpromazine (CPZ, an antipsychotic medication), (ii) cyclosporine A (CSA, an immunosuppressant medicine), (iii) erythromycin ethylsuccinate (EES, an antibiotic), (iv) glibenclamide (GBC, an oral anti-diabetic medication), (v) ketoconazole (KC, an antifungal medication), (vi) methyltestosterone (MTS, an anabolic–androgenic steroid), (vii) nifedipine (NFD, a calcium channel blocker), (viii) rifampicin (RIF, a macrolide antibiotic), and (ix) ticlopidine (TCP, an antiplatelet medication). These compounds are known to cause liver cholestasis via different pathophysiological mechanisms, indicating a number of molecular initiating events (MIEs) (i.e., transporter changes, hepatocellular changes and bile canalicular alterations) and key events (KEs) (i.e., inflammation, mitochondrial impairment, oxidative stress, endoplasmic reticulum (ER) stress and the simultaneously triggered adaptive response) driving the pathogenesis of DIC.

For the external test set retrieved from the National Center for Biotechnology Information Gene Expression Omnibus (GEO) (Barrett et al. 2013), less stringent criteria were applied. DIC-positive compounds were selected based on clinical case reports or peer-reviewed articles indicating cholestasis-inducing mechanisms. DIC-negative compounds included VITC, DMAN, RES, and the hepatoprotective QUE (Diabetes et al. 2012). Additionally, acetaminophen (APAP), a well-known hepatotoxin with distinct liver injury mechanisms, was labeled DIC-negative.

Only one DIC-positive compound, CSA, was shared between the training and validation sets. The selected compounds and their abbreviations are provided in Supplementary Table 1. The Supplementary Table 2 presents the mechanisms involved in the adverse effects of the DIC-positive compounds within the training set.

### Data collection and normalization

The training set utilized gene expression data from the Open TG-GATEs database, containing microarray-based profiles from in vitro cultured primary human hepatocytes (PHHs) and in vivo rat studies after treatment with over 150 compounds (Igarashi et al. 2015). The selected 18 compounds’ in vitro gene expression profiles were retrieved from PHHs treated at 2 time points (8 and 24 h) with 3 concentrations. The highest concentration defined the maximally tolerated dose with an 80–90% relative survival ratio. The middle- and high-dose levels had a ratio of 1:5.

For the external test set, transcriptome expression profiles in human hepatoma HepG2 cells were retrieved from published data (GEO accession number: GSE28878) (Magkoufopoulou et al. 2012) after exposure to the selected ten compounds and solvents for 12 and 24 h.

Gene expression profiles were measured using the Affymetrix GeneChip in both sets, and data were normalized using the robust multi-array average (RMA) method (*affy* package from R Bioconductor https://bioconductor.org/).

### Differential expression (DE) analysis

To identify DEGs upon treatment of PHHs with DIC or non-DIC compounds at each time point, statistical analyses were performed on the batch-corrected gene expression data using the *limma* package from R/Bioconductor. DEGs at each time point were defined as those transcripts with a |fold change (FC)|≥ 1.2 and Benjamini–Hochberg adjusted *p* value ≤ 0.05 in DIC-treated PHH relative to non-DIC-exposed cells. When analyzing the significance of the differential expression, two approaches were used, namely (i) directly comparing the differences of mean expression levels between the two groups using the batch-corrected gene expression values (rawExpression-derived DEGs: “DEGs”) and (ii) for each gene, the solvent controls were subtracted from the treated values. The batch-solvent-corrected gene expression values were used to investigate DEGs discriminating between DIC and non-DIC treatments (deltaChange-derived DEGs: “deltaDEGs”). Kyoto Encyclopedia of Genes and Genomes (KEGG) pathway enrichment analyses of the two DEG subsets performed using *KEGGgraph* package in R and Bioconductor. A *p* value ≤ 0.05 was considered statistically significant. Both the DEGs and deltaDEGs were extracted from the batch-corrected and the batch-solvent-corrected expression data for the following analyses.

### Machine learning models

We employed eight classification algorithms (logistic regression (LR), random forest (RF), Gaussian process (Gaussian), support vector machine (SVM), neural network multilayer perceptron classifier (NNMLPC), K-nearest neighbors (KNN), adaptive boosting (ADA), and extreme gradient boosting (XGBoost)) programmed in Python using the scikit-learn (sklearn) package (Pedregosa et al. 2011). Hyperparameter optimization and model training were performed on the training set (70% of the data) (Supplementary Table 3) using a fivefold grid-search cross-validation strategy. Feature importance was estimated using permutation-based feature selection.

To select features that are important for a model, permutation feature importance (PFI) scores for the full set of features were calculated. Features with nonzero positive permutation-based importance scores were selected and used in the following analysis. To further select the most significant features related to a compound’s potential to induce DIC, three wrapping algorithms available in *sklearn*, including two sequential search approaches (i.e., sequential forward (SFS) and backward (SBS) selection) (Rodriguez-Galiano et al. 2018) and RFE (Youssef et al. 2019), were adopted to select the optimal feature subsets containing the minimum number of genes.

To assess the models’ performance, we conducted 100-round, 5-fold cross-validation in the training sets and evaluated accuracy, area under the curve (AUC), sensitivity, specificity, positive predictive value (PPV), negative predictive value (NPV), and F1-scores. The feature subset with the least number of genes and the highest mean value across all evaluation metrics was considered the optimal feature set.

### External validation

The developed prediction model and DIC feature genes were tested on an external validation set with nine out of ten unseen compounds (Supplementary Table 1). The Gaussian and LR models, along with the identified relevant genes, were applied to the batch-corrected and batch-solvent-corrected validation sets, respectively. Accuracy, AUC, sensitivity, specificity, PPV, NPV, and F1-scores were calculated.

## Results

### Batch effect evaluation and removal

Technical variables in microarray-based gene expression studies, such as sample preparation and labeling, can introduce artifacts that obscure biological effects (Coppola 2011). The TG-GATEs dataset, containing data from multiple research organizations, may be affected by non-biological variables.

To address this, we built models using integrated transcriptional profiles derived from 18 compounds. These profiles were measured using six different lots of PHHs (CELL0020, 0030, 0040, 0050, 0060, and 0080) (Igarashi et al. 2015), and differences between the lots were considered a batch effect. Supplementary Fig. 1a reveals a batch effect associated with the cell lot number, with the CELL0030 array samples clearly separated from the rest. This effect was also evident in the hierarchical clustering dendrogram (Supplementary Fig. 2a). However, after applying batch correction, the effect was no longer present in the PCA (Supplementary Fig. 1b–f) or the hierarchical clustering outcome (Supplementary Fig. 2b). After correction, time point emerged as the most significant factor contributing to the expression data segregation. (Supplementary Fig. 1b).

### Outcomes of the DE analyses

*Limma* package (Ritchie et al. 2015) was used to obtain DEGs (FC ≥ 1.2; Benjamini–Hochberg adjusted *p*-value of ≤ 0.05) between DIC and non-DIC treatments. As time point became the variable capturing maximal variance in the batch-effect-corrected data, the DE analysis was conducted at the different time points.

Using the batch-corrected but solvent-uncorrected data, 133 and 69 genes were differentially expressed at 8 h and 24 h, respectively. The batch and solvent-corrected data yielded 66 and 209 DEGs at the 2 time points. Combining the results of the 2 time points, the rawExpression and deltaChange datasets, respectively, generated 174 (the “DEG” set) and 209 (the “deltaDEG” set) unique genes, with a total overlap of 46 between the 2 gene sets (Supplementary Fig. 3).

To gain further insight into the 2 sets of identified genes at the functional level, KEGG pathway analyses were performed for time-point-specific gene lists derived from both datasets (Supplementary Table 4). In total, the DEGs derived from the 8-h dataset resulted in 21 significant pathways, where the “Drug metabolism-cytochrome P450” (*p* value = 0.0082), “Metabolism of xenobiotics by cytochrome P450” (*p* value = 0.0114), and “Taurine and hypotaurine metabolism” (*p* value = 0.0126) related pathways appeared in the top 10 highest ranked KEGG results. It is worth mentioning that “Bile secretion” and “ABC transporters”-related pathways were also enriched, but the results were not significant (*p* value = 0.0723 and 0.3826, respectively). At 24 h, the DIC-associated DEGs only resulted in four significantly enriched KEGG pathways (“Protein processing in endoplasmic reticulum” with *p* value = 2.01E-07, “Influenza A” with *p* value = 0.0059, “Longevity regulating pathway-multiple species” with *p* value = 0.0348 and “Hepatitis C” with *p* value = 0.04229). Notably, “p53 signaling pathway” and “Bile secretion” were also enriched with marginally significant *p* values (*p* value = 0.061 and 0.065, respectively).

At the early time point, the genes on the deltaDEG list yielded fewer significant results than the DEG-produced results. At 8 h, four pathways (“Terpenoid backbone biosynthesis”, “Legionellosis”, “FoxO signaling pathway”, and “Biosynthesis of amino acids”) were significantly enriched as the deltaDEGs. Although the “Bile secretion” was also enriched, the result was not significant (*p* value = 0.3175). At the later time point, the deltaDEGs resulted in 12 significantly enriched pathways, including the “Porphyrin metabolism”, an activity that, once disrupted, could lead to cholestatic phenotype and oxidative stress that contribute to the development of hepatobiliary disease in patients (Casanova-Gonzalez et al. 2010; Smith and Foster 2018). Besides, a marginally significant enrichment in “Primary bile acid biosynthesis” has also been observed (*p* value = 0.069). In addition, two other cholestasis-relevant pathways, “Bile secretion” and “ABC transporters”, appeared, whereas the results were not significant (*p* value = 0.171 and 0.510, respectively).

### Model performance using PFI-selected features

The KNN method using 92 PFI-selected features achieved the highest mean predictive value (0.959) for the DEG set (Supplementary Table 5, section A). XGBoost and Gaussian with 18 and 39 features, respectively, also showed promising results with mean predictive values of 0.950 and 0.935, respectively. However, the ADA and RF models with 9 and 15 features, respectively, had suboptimal performance with mean predictive values below 0.9 and were not used in the following feature reduction steps. Although the NNMLPC model demonstrated good performance, it required over 100 features and was, therefore, not continued in the study due to the high computational demands of running the program.

For the deltaDEG set (Supplementary Table 5, section B), the SVMlinear model using 76 PFI-selected features had the highest mean predictive value (0.978), while LR and SVMlinear with 17 and 15 features, respectively, also showed promising results with mean predictive values of 0.959 and 0.906, respectively. Based on these results, three models established using DEGs (KNN, XGBoost, and Gaussian) or the deltaDEG set (LR, SVMlinear, and NNMLPC) were continued with the further feature reduction steps.

### Enhancing model performance through feature subset optimization via wrapper methods

When comparing the DEG-generated results (Supplementary Table 6, section A), Gaussian model performed best (SFS = 0.961, SBS = 0.96, RFE = 0.934) among KNN and XGBoost models for each feature subset (KNN: SFS = 0.961, SBS = 0.95 and RFE = 0.923; and XGBoost: SFS = 0.926, SBS = 0.919 and RFE = 0.917). The Gaussian model constructed using 17 features selected using the SBS method showed an optimal overall predictive performance (mean predictive value = 0.960). Although this combination gave a slightly lower result than the prediction outcomes produced using the Gaussian model and SFS-selected 30 features (mean predictive value = 0.961) and the KNN together with SFS-identified 24 features (mean predictive value = 0.960), the differences were not significant (Student’s *t* test *p* value = 0.318 and 0.814, respectively).

DeltaDEG results (Supplementary Table 6, section B) showed SVMlinear had the best performance for predicting DIC, but with a larger number of features. LR with a similarly small number of features outperformed NNMLPC (LR: SFS = 0.959, SBS = 0.962, RFE = 0.959, NNMLPC: SFS = 0.916, SBS = 0.915, RFE = 0.916). Gaussian model with 17 genes and LR with 13 features from feature elimination were further assessed using external test set.

### Results of external validation

To further evaluate generalizability, we assessed the performance of the two model-feature combinations using an external test set, including nine drugs previously unseen by the models. The Gaussian model with 17 selected features performed poorly with a mean predictive value of 0.471 (accuracy: 0.475, AUC: 0.487, sensitivity: 0.400, specificity: 0.550, PPV: 0.471, NPV: 0.478 and F1-score: 0.432). Out of 120 samples in the batch-corrected external test set, 28 and 35 were incorrectly predicted at 12-h and 24-h time points, respectively.

Using the batch- and solvent-corrected external data (60 samples), the LR model with deltaDEG-derived 13 genes yielded an over 0.706 mean predictive value (accuracy: 0.700, AUC: 0.716, sensitivity: 0.767, specificity: 0.633, PPV: 0.676, NPV: 0.731, and F1-score: 0.719). However, 18 of the 60 samples were classified as having a toxicity level different from the expected ground truth, including 9 samples each measured at 12-h and 24-h time points. Notably, the LR model accurately predicted samples generated from 3 of the 5 DIC-positive compounds after 12-h exposures and correctly recognized 3 DIC-positive and 1 DIC-negative drugs among all the 24-h cases, implying that the 13 deltaDEG-derived features contain common information shared by cholestasis-inducing compounds.

### The biological interpretation of the identified DIC signature

The previously published AOP network on DIC offers a conceptual framework that consolidate existing knowledge and research findings related to the molecular mechanisms that contribute to the development of intrahepatic cholestasis (Gijbels et al. 2020; Vinken et al. 2013). To identify potential targets for hazard characterization, we analyzed the biological functions and the direction of expression changes of the genes that were significantly differentially expressed in DIC-treated samples compared to non-DIC-treated samples, and were selected by the LR model to distinguish between the two groups.

Table 1 summarizes the general functions of these genes, which were grouped into various biological activities. We found that 9 of 13 genes were associated with KEs known to be associated with the development of cholestasis, including BA synthesis, bile flow disruption, oxidative stress, inflammation, ER stress, and apoptosis. In addition to genes associated with deteriorative response-related key events, this DIC signature also includes genes linked to the adaptive response initiated to counteract the BA accumulation (i.e.,* TSKU* and *ALAS1*). The putative functions of two less investigated genes (i.e.,* SLC16A3* and *VSIG10L*) were found to correlate with previously discovered key events involved in the development of cholestasis, specifically bile flow disruption, and autophagy.

**Table 1:** General functions of genes identified using the machine learning strategy developed in this study

Gene symbol	Gene name	General function
*ALAS1*	5'-aminolevulinate synthase 1	Adaptive response*
*LMAN1*	Lectin, mannose binding 1	ER stress*
*MMP3*	Matrix metallopeptidase 3	ECM remodeling
*NDUFA4L2*	NDUFA4 mitochondrial complex associated like 2	Oxidative stress*; Apoptosis*
*PMP22*	Peripheral myelin protein 22	Apoptosis*
*PPDPF*	Pancreatic progenitor cell differentiation and proliferation	Apoptosis*
*SEMA6C*	Semaphorin 6C	Apoptosis*
*SLC16A3*	Solute carrier family 16 member 3	*Bile flow disruption*
*SLC9A3R2*	NHERF family PDZ scaffold protein 2	BA synthesis*
*TM4SF1*	Transmembrane 4 L six family member 1	Apoptosis*
*TMPRSS11D*	Transmembrane serine protease 11D	Inflammation*
*TSKU*	Tsukushi, small leucine rich proteoglycan	Adaptive response*; BA synthesis*
*VSIG10L*	V-Set and immunoglobulin domain containing 10 like	*Oxidative stress*; autophagy

*BA* bile acid, *ER* endoplasmic reticulum

*Biological activity that overlaps with the known key event associated with the development of chemical-induced cholestasis, italic general function: putative function

**Fig. 2:**
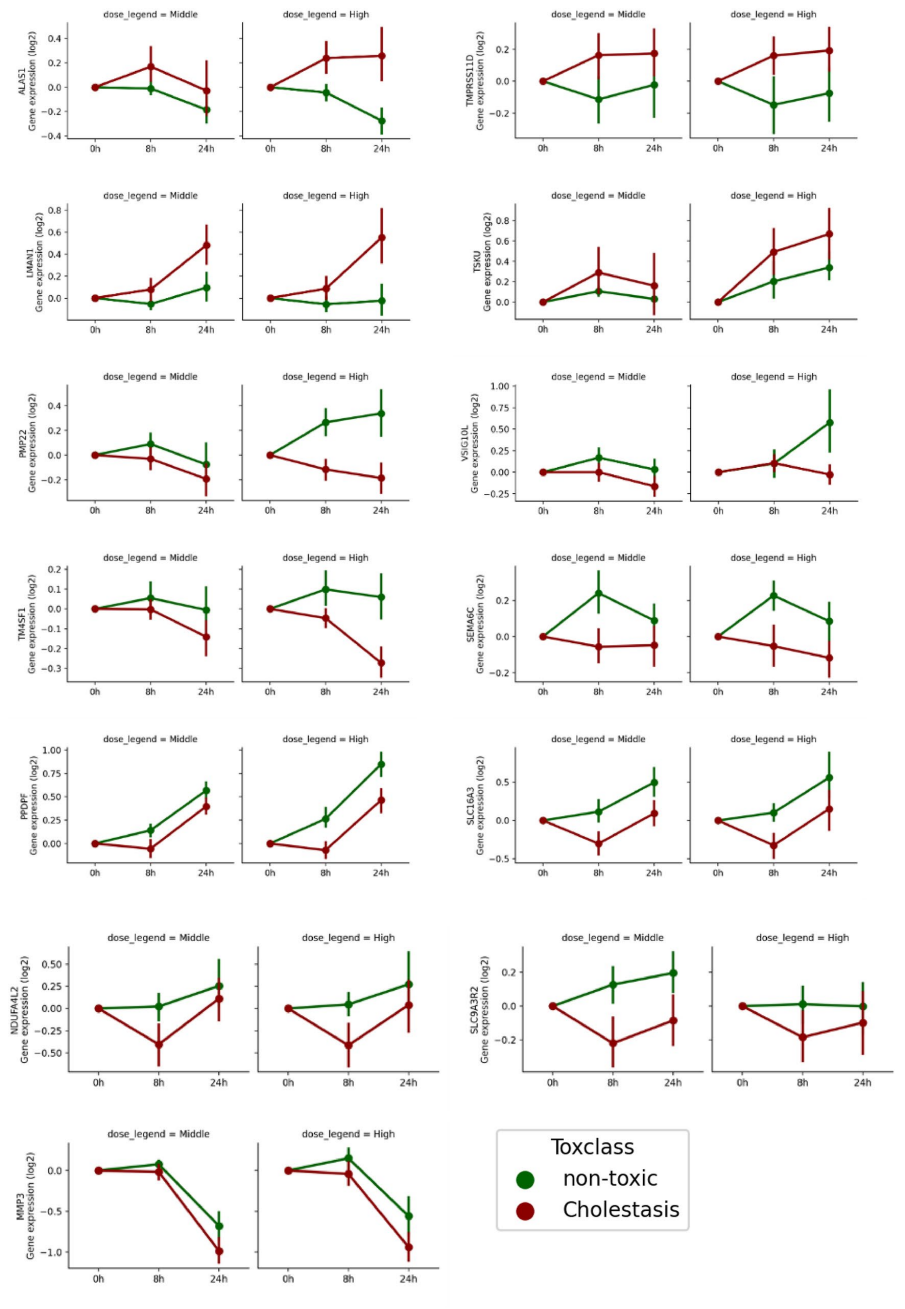
Time-course changes of the differentially expressed genes. Gene expression levels were measured at different time points after treatment, and the changes in expression levels were compared to the baseline values, which were defined as the average expression levels in the 8-h solvent control samples. The expression levels are shown in relative units, with higher values indicating higher expression levels. The expression levels in the DIC-treated samples are shown in red, while those in the non-DIC-treated samples are shown in green (color figure onlie)

To determine the direction of expression changes for individual genes, we used the training dataset and established the expression levels following 8 h of solvent treatment as the baseline values, and plotted time-course transcriptome changes for genes identified by the LR model as important for distinguishing between DIC and non-DIC-treated samples (Fig. 2). Out of the 13 identified genes, 4 (*ALAS1*, *TMPRSS11D*, *LMAN1*, and *TSKU*) consistently showed elevated expression levels in DIC-treated samples compared to non-DIC-exposed specimens. Conversely, the other nine genes had higher expressions in non-DIC-treated samples. *PMP22*, *VSIG10L*, *TM4SF1*, and *SEMA6C* were induced after high-dose non-DIC treatments, but suppressed over time after exposure to DIC compounds. *PPDPF*, *SLC16A3*, *NDVFA4L2*, and *SLC9A3R2* showed continued increases in expression in non-DIC-treated cells, whereas their expression was first inhibited at the early time point but then upregulated in DIC-treated cells. *MMP3* showed a similar declining trend in expression after treatment with both DIC and non-DIC compounds, with a more intense drop in expression in DIC-exposed cells.

## Discussion

Predicting drug-induced cholestasis (DIC) based solely on bile acid transporter malfunctions has had limited success. Identifying a DIC signature using microarray data and machine-learning-based feature selection approaches can offer a data-driven method to distinguish toxicants (Mahendran et al. 2020). This study aimed to build a prediction model using high-dimensional microarray data and ML-based feature selection approaches to improve dimensionality reduction and identify a translatable DIC signature. Such a signature could aid in in vitro DIC prediction, facilitating early detection of this chemical-induced toxicity.

DE analysis identified genes revealing the mechanisms underlying DIC in PHHs by comparing transcriptomic patterns of cells exposed to DIC and non-DIC compounds using batch-corrected and solvent-corrected training data. Each comparison yielded distinct subsets of DEGs (174 and 256 genes). The DEG set showed more significant pathways related to toxic responses and DILI development, especially at early time points, while the deltaDEG set yielded fewer significant results and limited hepatotoxicity-relevant pathways. However, the optimized Gaussian model on DEGs did not perform as well as the LR model on deltaDEGs in the external validation set. A possible explanation for this discrepancy could be that, in addition to the size of the gene set, gene set composition might influence enrichment analysis sensitivity. Each individual gene may have varying degrees of association with the specified trait that the set is designed to encapsulate, and the sensitivity of the analysis can be affected by the mixture of strongly associated and weakly associated genes in the set (Davies et al. 2010). These findings suggest that, while the removeBatchEffect function corrected the batch effect in the training set, other factors such as the solvent factor may still introduce noise into the data. Additional solvent correction may reduce noise and enhance transcriptomic accuracy. Therefore, this study highlights that a careful gene set selection is crucial for robust enrichment analysis.

DE analysis often generates numerous correlated candidate genes, leading to redundant information and reduced translatability for laboratory testing (Abbas and El-Manzalawy 2020) and lowered translatability of the DE findings for high-throughput laboratory testing. To address this, a permutation-based approach was employed to refine the results that estimate feature relevance by measuring changes in model performance upon permuting feature vectors (Altmann et al. 2010). This method avoids bias introduced by Gini importance and coefficient-based approaches, which may overlook feature interactions or be affected by multicollinearity and outliers in the data (Altmann et al. 2010) (Midi et al. 2010; Strobl et al. 2007) (Park and Liu 2011).

ML models were assessed for predicting outcomes based on transcriptomic data. Internal validation yielded promising results for DEG and deltaDEG sets, but the Gaussian model performed poorly on the batch-corrected external set. Despite the related 17 features showing mechanistically plausible functions (Supplementary Table 7), poor performance of the Gaussian model might be due to the structural differences between the training and external sets. However, the LR model, using 13 genes, achieved a mean predictive value of 0.71 in the batch- and solvent-corrected external validation dataset. The identified DIC 13-gene signature exhibited mechanistically plausible functions related to key events within the cholestasis AOP network (Gijbels et al. 2020; Vinken et al. 2013), such as bile flow disruption, inflammation, ER stress, oxidative stress, autophagy, apoptosis, and adaptive response. These findings suggest that removing solvent controls improved the generalizability of the model, producing less noisy transcriptomic profiles and a structurally similar external set. The 13-gene signature demonstrated a broad functional impact in DIC-related pathways.

In detail, we observed a significant decrease in the expression of *SLC9A3R2*, a member of the Na + /H + exchanger family and a PDZ scaffolding protein, in PHHs exposed to DIC at an early time point. *SLC9A3R2* is involved in various physiological activities, such as transepithelial Na + and water absorption, acid–base and fluid volume homeostasis, and regulation of membrane receptors and transport proteins (Xu et al. 2018). One of the transport proteins regulated by *SLC9A3R2* is the scavenger receptor class B type 1 (SR-B1), which is responsible for converting hepatic HDL-cholesteryl ester to BAs (Lu et al. 2017). Previous studies have shown that SLC9A3R2, together with NHERF1, regulates SR-B1 protein levels by promoting its degradation (Lu et al. 2017).For this reason, the decreased expression of *SLC9A3R2* due to DIC exposure may inhibit the degradation of SR-B1, leading to increased production of BAs in PHHs. In contrast to the upregulation observed in non-DIC samples, DIC exposure downregulated expression of *SLC16A3*, a gene that encodes monocarboxylate transporter 4 (*MCT4*), a proton-coupled transmembrane protein responsible for transporting BAs and organic acids across cell plasma membranes (Schumann et al. 2020). Although the relationship between *SLC16A3* and cholestasis pathogenesis is unclear, recent research has identified this gene as a potential prognostic biomarker related to intrahepatic cholangiocarcinoma cell reprogramming (Dong et al. 2022). Hence, we speculate that the reduced *SLC16A3* expression induced by DIC exposure, at least in early time points, may lead to altered BA metabolism or transport in PHHs, potentially resulting in disrupted BA homeostasis and the accumulation of noxious BAs in liver cells. The observed changes in the expression of the two genes in response to DIC exposure may have affected BA metabolism and transport, resulting in the accumulation of noxious BAs and activation of a deteriorative response in the liver.

The initial stages of the deteriorative response to DIC involve inflammation and mitochondrial impairment, which can result in oxidative stress and in turn trigger ER stress in the liver ^4^. The expression of *TMPRSS11D* showed a dose-dependent and/or time-dependent increase in response to DIC exposure. The protein product of this gene, also known as human airway trypsin-like protease (HAT), has been reported to promote pro-inflammatory responses in epithelial cells by enhancing cytokine production and recruiting inflammatory cells (Menou et al. 2017). This suggests that the induction of *TMPRSS11D* may play a role in amplifying the inflammatory response in PHHs exposed to cholestatic compounds. *NDUFA4L2* encodes an electron transport chain complex I subunit located in mitochondria, which acts as an antioxidant to regulate cell survival by restraining reactive oxygen species (ROS)-mediated apoptosis (Meng et al. 2019). After DIC treatment, *NDUFA4L2* expression declined early on and remained lower compared to non-DIC samples. Previous studies have shown that inactivation of NDUFA4L2 led to ROS accumulation and increased apoptosis in hepatocellular carcinoma cells (Lai et al. 2016), while upregulation of *NDUFA4L2* attenuated oxidative stress associated with intervertebral disc degeneration (Liu et al. 2021). The observed decrease in *NDUFA4L2* expression in response to DIC exposure, therefore, may result in enhanced oxidative stress and increased apoptosis in treated cells. In addition to the changes in the expression of genes of interest, DIC treatment also induced a significant increase in *LMAN1* expression, which encodes ERGIC-53, a protein located in the ER-Golgi intermediate compartment. It has been shown that ER stress can regulate the transcriptional expression of *LMAN1*, which carries out functions in the post-ER compartments of the secretory pathway (Renna et al. 2007). Thus, the increase in *LMAN1* expression observed after the DIC exposure could indicate the possibility of ER stress activation. Our findings imply that DIC-exposure-induced changes in the expression of *TMPRSS11D*, *NDUFA4L2* and *LMAN1* genes may contribute to inflammatory response amplification, oxidative stress enhancement, and ER stress activation during initial stages of cholestasis, which can lead to cell death, another KE in the AOP network for DIC.

In addition to *NDUFA4L2*, the expression of four other apoptosis regulators was affected by the DIC compound exposure, which could potentially contribute to cell death during the initial stages of cholestatic liver injury. Specifically, the expression of *TM4SF1*, a gene encoding a transmembrane protein, was found to be repressed in a time- and dose-dependent manner by DIC treatment. Previous studies have demonstrated that *TM4SF1* exerts an anti-apoptotic effect on cells, such as human hepatoma HepG2 cells (Huang et al. 2016) and human gastric cancer cells (Wei et al. 2018). Consequently, the suppressed expression of *TM4SF1* after exposure to DIC compounds could promote apoptosis in PHHs, contributing to the observed adverse effect. *SEMA6C* expression was elevated in non-DIC-treated cells after high-dose treatments but decreased in cholestatic compound-exposed cells. *SEMA6C* encodes an axon guidance factor that may function as a tumor suppressor by inhibiting the AKT/GSK3 signaling pathway, which in turn activates the intrinsic mitochondrial apoptotic event through the PI3K/Akt signaling axis (Hung et al. 2022). The increased expression of *SEMA6C* after non-DIC treatments could, therefore, potentially prevent cells from undergoing apoptosis in response to different stimuli, but this protective effect may not be present in DIC-treated cells. Furthermore, our study revealed upregulated expression of *PMP22* and *PPDPF* in non-DIC-treated PHHs in a time- and dose-dependent manner, but their expression was relatively lowered in DIC-exposed cells. *PMP22* and *PPDPF*, which are highly expressed in bile canaliculi (Notterpek et al. 2001) and human hepatocytes (Ma et al. 2021), have been shown to have anti-apoptotic effects in various cell types, such as lung (Yun et al. 2022), gastric (Hou et al. 2021), and neural cells (Sancho et al. 2001). As a result, the relatively lowered expression of these two genes in DIC-exposed cells may contribute to increased apoptosis. It is interesting to note that peroxisomal membrane protein encoded by *PMP22* (Fan et al. 1996) is considered a constituent of intercellular junctions in epithelia (Notterpek et al. 2001), suggesting a potential role in maintaining tight junction integrity in hepatocytes.

Alongside the deteriorative response, an adaptive response is triggered to counteract the accumulation of BAs by activating nuclear receptors, such as the constitutive androstane receptor (CAR) and farnesoid X receptor (FXR), which regulate the expression of genes involved in BA homeostasis to alleviate cholestasis ^4^. We observed that exposure to DIC compounds induced the expression of the *TSKU* and *ALAS1* genes in a dose-dependent and/or time-dependent manner. As a target gene of CAR, *TSKU* plays a crucial role in BA synthesis (Zollner and Trauner 2009). Its protein product can reduce cholesterol efflux and negatively regulate cholesterol conversion to BAs in rodents' livers (Mouchiroud et al. 2019). Increased *TSKU* expression after high-dose DIC treatments may mitigate BA toxicity via CAR-mediated adaptive responses. Similarly, FXR, another BA-activated nuclear receptor, is a direct regulator of human hepatic *ALAS1* (Zollner and Trauner 2009). *ALAS1* encodes a mitochondrial enzyme that catalyzes the rate-limiting step in heme synthesis in the liver (Maestro et al. 2021) and is critical in facilitating BA detoxification by providing sufficient heme for newly synthesized apocytochromes (Peyer et al. 2007). The induction of *ALAS1* expression after high-dose DIC exposure may, therefore, suggest an adaptive response to cope with BA accumulation in PHHs.

Our analysis also revealed distinct expression patterns for *MMP3* and *VSIG10L* in DIC and non-DIC compound treatments. Among these genes, *MMP3* showed a remarkable reduction in expression levels in both DIC and non-DIC treatments, with a more pronounced repression in DIC-exposed cells. *MMP3* encodes a matrix metalloproteinase, which is known to play a critical role in maintaining extracellular matrix (ECM) homeostasis by breaking down *MMP3*-sensitive ECM components in physiological and pathological processes, such as liver fibrosis (Juran et al. 2011; Miyahara et al. 2000). Interestingly, elevated *MMP3* expression has been observed in patients with primary biliary cholangitis (PBC), a chronic cholestatic liver disease that often progresses to cholestasis, fibrosis, cirrhosis, and liver failure (Bauer and Habior 2022). This suggests that strong suppression of *MMP3* expression induced by DIC exposure may lead to an imbalance between ECM production and degradation, thereby increasing the risk of disease progression and exacerbating liver injury over prolonged treatment. This finding provide new insights into the biological mechanisms underlying the development of DIC and highlight the importance of *MMP3* in maintaining liver homeostasis. *VSIG10L* is a poorly characterized gene, but studies have indicated a dual nature of its expression in relation to cancer development. While downregulated in esophageal adenocarcinoma, it was upregulated in lung squamous cell carcinoma (Zhou et al. 2022). *VSIG10L* shares structural similarity with *VSIG10* (Zhou et al. 2022), a gene regulated by NFE2 like BZIP transcription factor 2 (*NFE2L2*) (Qian et al. 2015), which protects against oxidative stress (Wolf et al. 2016) and activates autophagy in epithelial cells (Chang et al. 2022). As such, *VSIG10L* may also have antioxidant properties, which may explain its upregulation in non-DIC-treated cells. However, the exact role of *VSIG10L* in cancer development or other diseases and its transcriptional changes remains unclear.

Overall, the feature selection pipeline presented in our research has great potential for improving the accuracy and reliability of transcriptomic profiling and gene set enrichment analysis. The gene signature identified using the pipeline sheds new light onto the biological mechanisms of cholestasis development and identifies potential targets for intervention and hazard characterization.

In this study, we developed a data-driven approach for identifying a transcriptomic signature that can predict DIC. The results underscore the importance of validating prediction models on independent datasets, as models that perform well during internal validation may not generalize well to different datasets that use unique compounds or measurement techniques. Applying a solvent-correction step to transcriptomic data can reduce bias and confounding effects, making the data more reliable and translatable to other data sets. By selecting model compounds that induce DIC through diverse toxic mechanisms, we identified a gene signature that has potential applications beyond the compounds used in this study. The identified features have biologically interpretable functions, mechanistically anchored in an AOP network, and provide new insights into molecular and cellular behavior processes during DIC development, making them valuable tools for understanding and predicting toxicological responses.

## Supplementary Information

Below is the link to the electronic supplementary material.

Supplementary file1 (DOCX 2460 KB)

## Data Availability

The data used for composing the training set that support the findings of this study are available in Open TG-GATEs database with the identifier https://dbarchive.biosciencedbc.jp/data/open-tggates/LATEST/Human/in_vitro/. The external test data were deposited into the Gene Expression Omnibus database under accession number GSE28878 and are available at the following URL: https://www.ncbi.nlm.nih.gov/geo/query/acc.cgi?acc=GSE28878 (ref (Magkoufopoulou et al. 2012)).
