# Supplementary material for: Unraveling the mechanisms underlying drug-induced cholestatic liver injury: identifying key genes using machine learning techniques on human in vitro data sets
Source: Arch Toxicol. 2023 Aug 21;97(11):2969–81. doi: 10.1007/s00204-023-03583-4 (PMC10504391; doi:10.1007/s00204-023-03583-4)
Supplement: Supplementary file 1 — Supplementary file1 (DOCX 2460 KB) [file 204_2023_3583_MOESM1_ESM.docx]

## Additional Methods:

### Training data preprocessing

A total of 19,898 annotated probe sets on the Human Genome U133 Plus 2.0 with official gene symbols were retrieved using *biomaRt*, an R/Bioconductor package. To facilitate biological interpretation of the DIC features in the later stage, only human-rat orthologs, consisting of 10,265 genes, were used in the following analyses. Low-variance expressed genes, defined as genes whose expression variability across all samples fell in the bottom decile of the genome-wide expression variance distributions, were removed.

To identify sources of unwanted variation, such as expression heterogeneity, batch effects or other sources of bias, in the dataset, the overall pattern of the expression distribution of the remaining genes was evaluated using principal component analysis (PCA) and hierarchical cluster analysis (HCA), utilizing the R functions *prcomp* (with the ‘center’ option set to TRUE) and *hclust* (calculating the pairwise Euclidean distance between samples), respectively. The 2-dimensional PCA plot was generated using the R function *autoplot*. To visualize the batch effect, dendrogram was colored by the batch factor. The batch effect was corrected using the *removeBatchEffect* function (default settings) in *limma* package (v3.42.9) implemented in the R. The batch-corrected data were used in the downstream analyses.

### Data preparation for machine learning

A total of 108 and 72 positive-negative training instances were obtained in the 2 subsets of the training data, respectively. For both training subsets, data with an evenly balanced distribution of positive and negative samples were randomly extracted from the dataset. As listed in Supplementary Table 2, 70% of the data were used as the training dataset for hyperparameter tuning and training, and the remaining 30% were used as the testing dataset to evaluate the performance of the models. The data separation was achieved using the *Scikit-Learn (sklearn)*’s *train_test_split* method.

### Model selection based on classification results using all DEG feature sets

To select potential models for subsequent analyses, all untuned models with default hyperparameters were tested on the DEG and deltaDEG sets to predict binary toxicity outcomes (Supplementary Figure 2). Using the 174 DEG set for prediction, the worst performing model was the dummy classifier (mean predictive value=0.539). As such, 6 models, namely NNMLPC, RF, XGBoost, Gaussian, ADA, and KNN, resulted in mean predictive values over 0.9 (i.e. the mean value in all 7 metrics), with relatively small variances across individual metrics. The 2 linear models, LR (mean predictive value=0.673) and SVMlinear (mean predictive values=0.616), provided suboptimal results (Supplementary Figure 2a).

When applying the algorithms to the 257 deltaDEGs, the NNMLPC achieved the highest mean predictive value (0.967), followed by LR and SVMlinear (mean predictive values of 0.946 and 0.938, respectively. As expected, the dummy classifier had the worst performance (mean predictive value=0.506). Although the mean predictive values produced by other models appeared to be within an acceptable range (from 0.859 to 0.921), we observed large differences across individual evaluation metrics for these models (Supplementary Figure 2b).

Based on our findings, we selected 6 models (i.e., NNMLPC, RF, XGBoost, Gaussian, ADA, and KNN) and three models (i.e., NNMLPC, LR, and SVMlinear) for the DEG and deltaDEG sets, respectively, for subsequent model tuning and feature selection.

### Evaluation metrics

The following evaluation metrics were used to evaluate the performance of the built classifiers:

Accuracy = (true positive (TP) + true negative (TN)) / (TP + TN + false positive (FP) + false negative (FN))

Sensitivity = TP/(TP+FN)

Specificity = TN/ (TN+FP)

PPV = 100xTP/(TP+FP)

NPV = 100xTN/(FN+TN)

F1-score = 2 TP / 2 TP + FP + FN

## Supplementary Figures and Tables

| a | 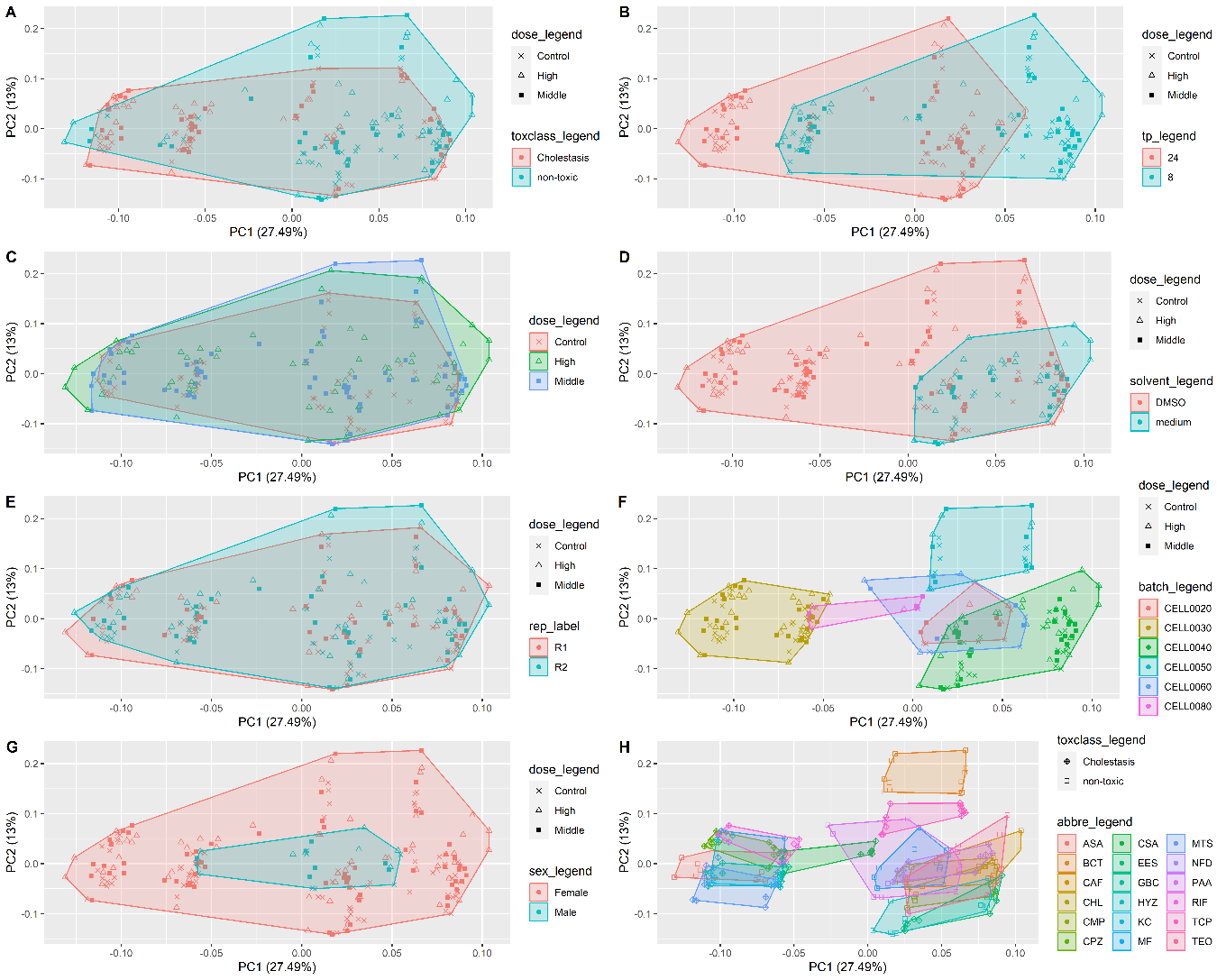 |
| --- | --- |
| b | 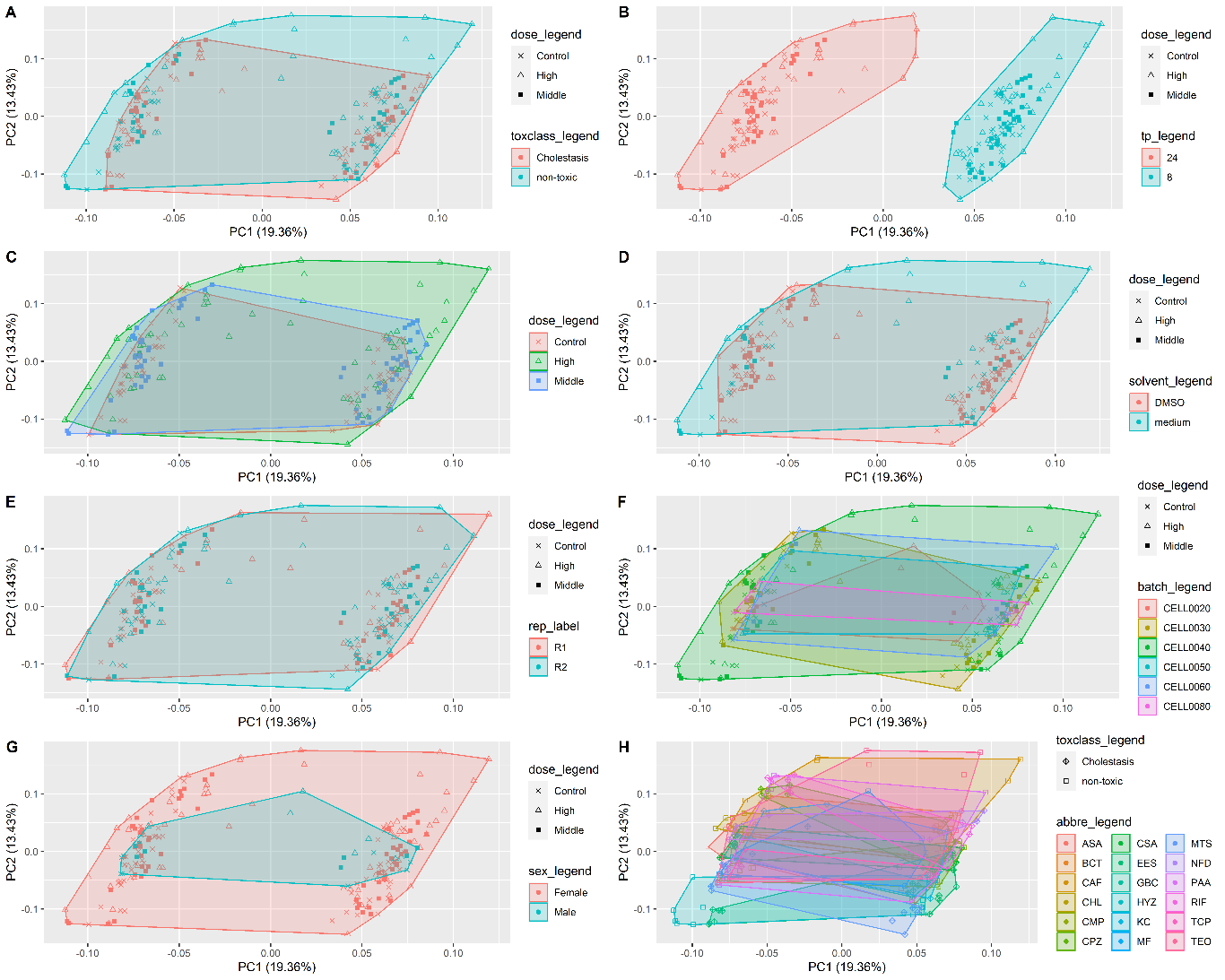 |
| **Supplementary Figure 1.** Batch correction reveals reduced batch effects and enhanced clustering based on time point. (a) PCA plots of the first 2 principal components for the uncorrected data, showing distinct groups corresponding to the 6 different cell lots. (b) PCA plots of the batch-corrected data after applying the removeBatchEffect functions, demonstrating improved clustering based on time point. Two distinct clusters emerge, representing the 8 and 24-hour time points. | |

| a | 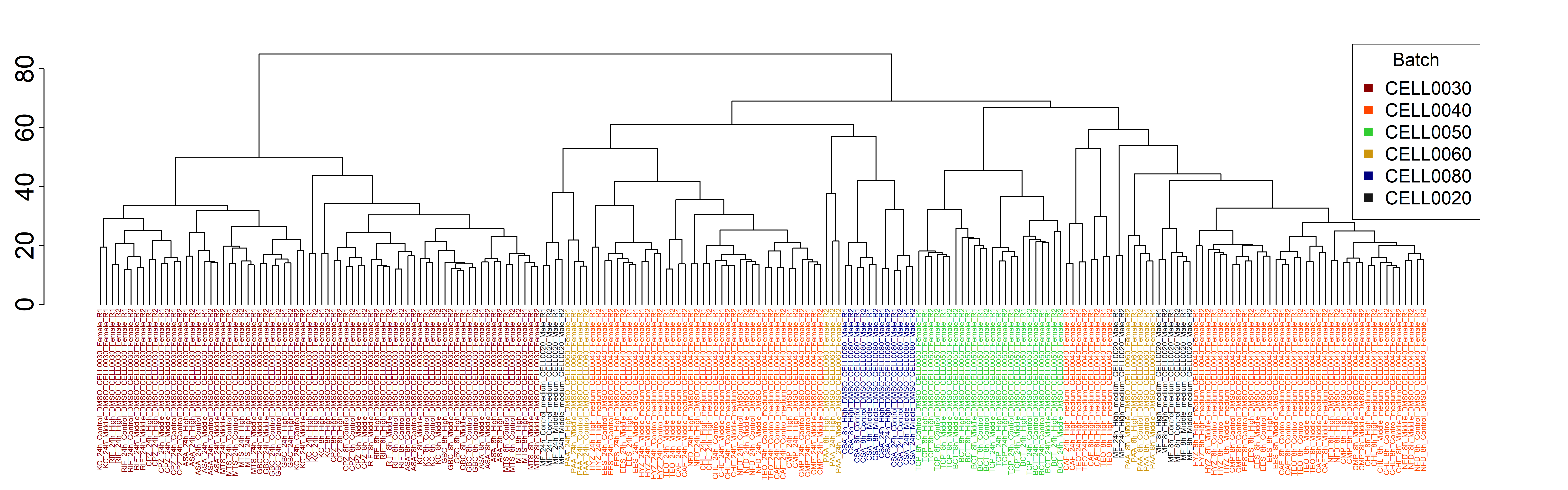 |
| --- | --- |
| b | 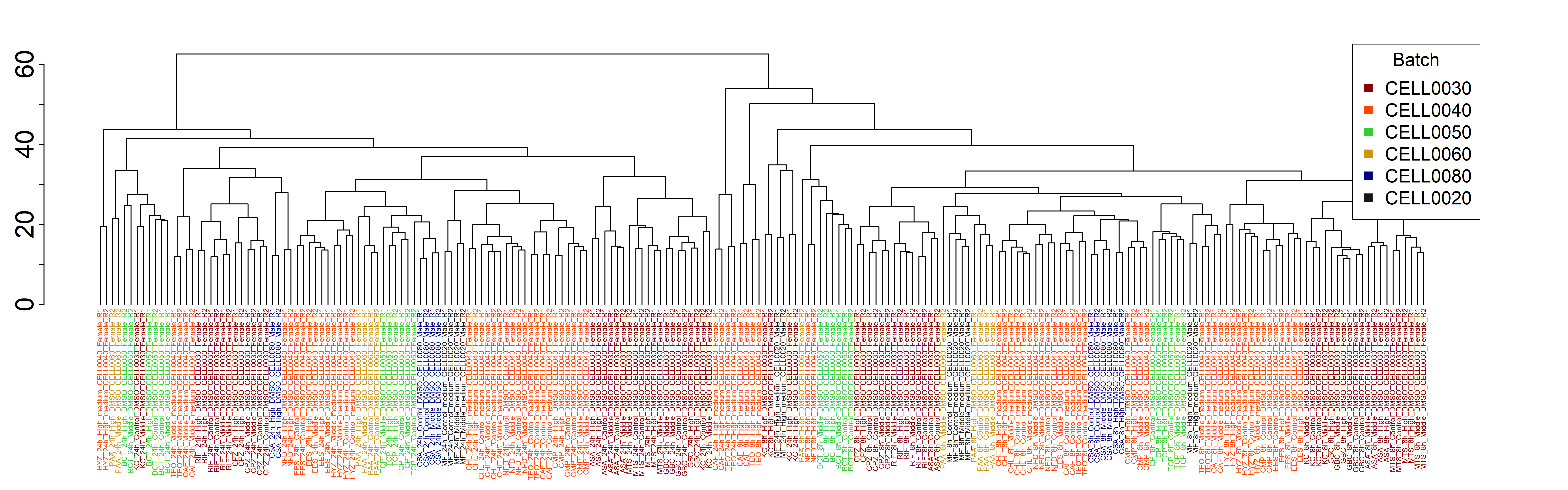 |
| **Supplementary Figure 2**. The effects of using PHHs from different lots on the data and how these effects can be reduced using the *limma* package's *removeBatchEffect* function. Hierarchical clustering of a) uncorrected and b) batch-corrected data. Colors indicate the batches of cells (information of cell lots). a) shows the clustering of uncorrected data, which forms two main clusters reflecting samples measured using cells from CELL0030 and from other lots. This indicates the presence of batch effects, which can confound the analysis of differential gene expression. b) shows the clustering of batch-corrected data after applying the *removeBatchEffect* function. The batch effect is no longer present in the hierarchical clustering outcome, suggesting that the *removeBatchEffect* function is an effective tool for reducing batch effects and improving the accuracy of differential gene expression analysis. | |

| a | 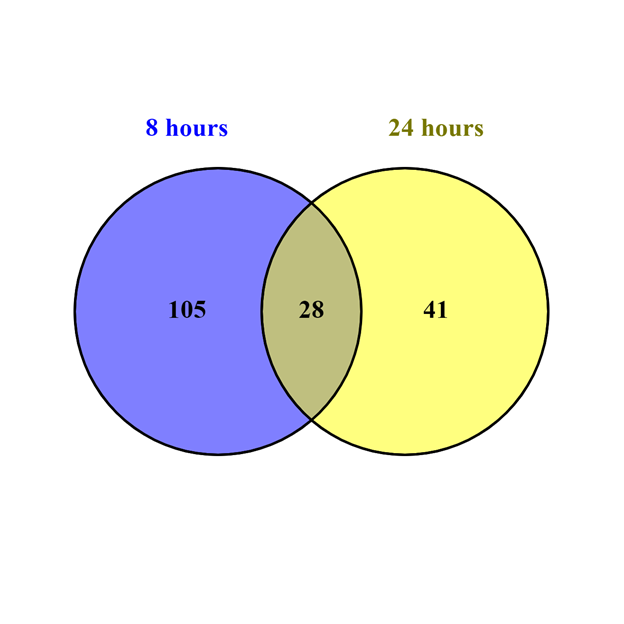 | b | 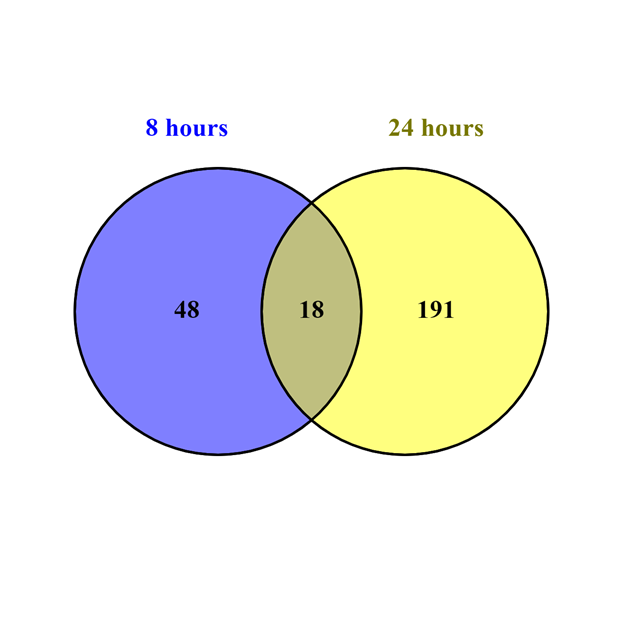 | c | 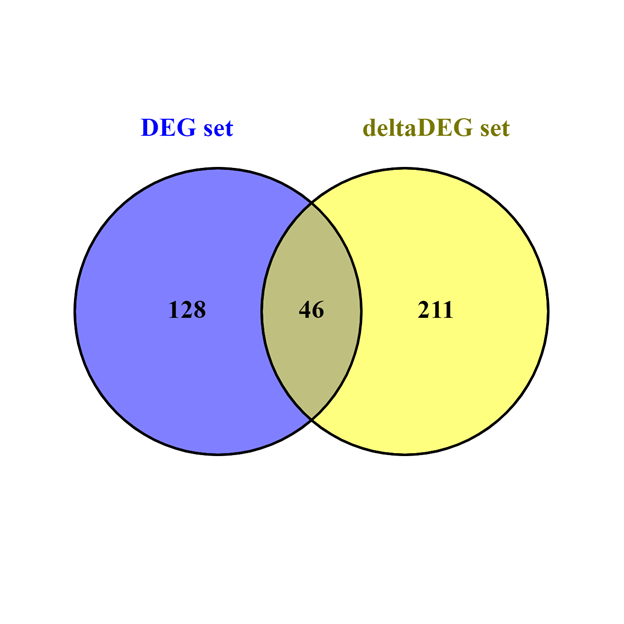 |
| --- | --- | --- | --- | --- | --- |
| **Supplementary Figure. 3** Venn diagram for differentially expressed genes. Venn diagrams of DEGs derived from (a) the batch-corrected but solvent-uncorrected data (the “DEG” set) and (b) the batch and solvent-corrected data (the “deltaDEG” set). (c) Comparison of the DEGs obtained from merging the lists at the two time points with the deltaDEGs. A total of 46 genes were found to be common between the two lists, demonstrating their shared significance. | | | | | |

| a | 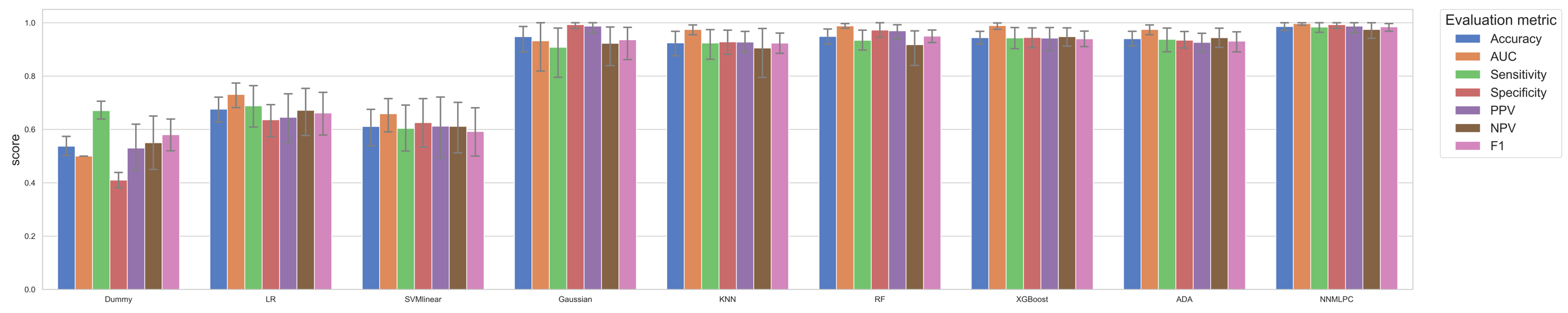 |
| --- | --- |
| b | 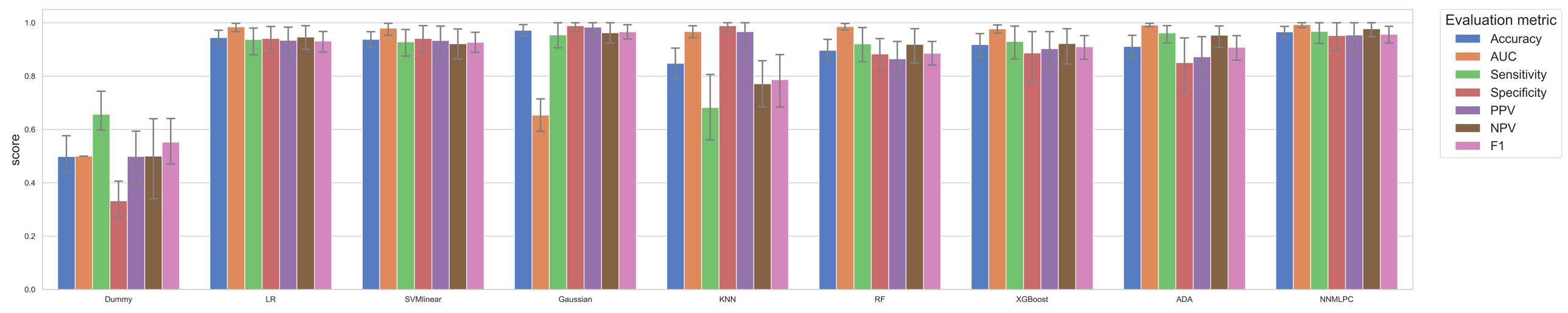 |
| **Supplementary Figure 4**. Selection of model based on various evaluation metrics. The values for different metrics were calculated based on a) DEGs and b) deltaDEGs using different untuned classification algorithms, namely LR, SVMlinear, SVMrbf, Gaussian, KNN, RF, ADA, XGBoost and NNMLPC, and a dummy classifier. | |

| **Supplementary Table 1.** Compounds used in the training and external test sets. | | | | | |
| --- | --- | --- | --- | --- | --- |
| Compound Name | Abbreviation | Cas No. | Compound Name | Abbreviation | Cas No. |
| Section A: Training set | | | | | |
| Aspirin | ASA | 50-78-2 | Chlorpromazine | CPZ | 50-53-3 |
| Bucetin | BCT | 1083-57-4 | Cyclosporine A | CSA | 59865-13-3 |
| Caffeine | CAF | 58-08-2 | Erythromycin ethylsuccinate | EES | 1264-62-6 |
| Chlorpheniramine | CHL | 132-22-9 | Glibenclamide | GBC | 10238-21-8 |
| Chloramphenicol | CMP | 56-75-7 | Ketoconazole | KC | 65277-42-1 |
| Hydroxyzine | HYZ | 68-88-2 | Methyltestosterone | MTS | 58-18-4 |
| Metformin | MF | 657-24-9 | Nifedipine | NFD | 21829-25-4 |
| Phenylanthranilic Acid | PAA | 91-40-7 | Rifampicin | RIF | 13292-46-1 |
| Theophylline | TEO | 58-55-9 | Ticlopidine | TCP | 55142-85-3 |
| Section B: External test set | | | | | |
| Acetaminophen | APAP | 103-90-2 | Cyclosporine A | CSA | 59865-13-3 |
| D-Mannitol | DMAN | 69-65-8 | Diethylstilbestrol | DES | 56-53-1 |
| Quercetin | QUE | 117-39-5 | 17beta-estradiol | E2 | 50-28-2 |
| Reserpine | RES | 50-55-5 | 2,3,7,8-Tetrachloro dibenzo-p-dioxin | TCDD | 1746-01-6 |
| L-Ascorbic acid | VITC | 50-81-7 | Wy-14643 | WY | 50892-23-4 |
| Non-DIC and DIC compounds. Section A shows the compounds used in the training set. Section B contains the compounds used in the external test set. Compounds in bold are shared by the 2 datasets. | | | | | |

| **Supplementary Table 2.** Mechanisms underlying the selected DIC-positive compounds. | | |
| --- | --- | --- |
| Mechanism | Compound abbreviation | Reference |
| Changes in the expression and/or activity of multiple BA transporters | CPZ | (Hendriks et al. 2016; Wu et al. 2022) |
|  | CSA | (Sharanek et al. 2014) |
|  | EES | (Morgan et al. 2010) |
|  | GBC | (Ni et al. 2016) |
|  | KC | (He et al. 2015; Jemnitz et al. 2010; Ni et al. 2016; Zhang et al. 2016) |
|  | MTS | (Vatakuti et al. 2017) |
|  | NFD | (Dawson 2011; Kock et al. 2014; Kolaric et al. 2019) |
|  | RIF | (Amor et al. 2018; Guo et al. 2015; Lau et al. 2007; Mita et al. 2006) |
|  | TCP | (Chatterjee et al. 2014)) |
| compromising cytoskeleton architecture | CSA | (Sharanek et al. 2014) |
| disrupting tight junctions and cell membrane integrity | CSA | (Sharanek et al. 2014) |
| initiating inflammatory responses) | CPZ | (Hendriks et al. 2016) |
|  | CSA | (Nsengimana et al. 2022) |
| altering bile canalicular dynamics | CPZ | (Sharanek et al. 2016) |
|  | CSA | (Sharanek et al. 2014) |
| blocking efflux of BAs | EES | (Jazaeri et al. 2021; Kostrubsky et al. 2003) |
| inhibiting BA uptake | GBC | (Horikawa et al. 2003) |
| triggering oxidative stress | CPZ | (Hendriks et al. 2016; Wu et al. 2022) |
|  | CSA | (Nsengimana et al. 2022; Sharanek et al. 2014) |
|  | RIF | (Xu et al. 2020; Yew et al. 2018) |
|  | TCP | (Chatterjee et al. 2014; Tolosa et al. 2012) |
| ER stress | CSA | (Sharanek et al. 2014) |
|  | MTS | (Vatakuti et al. 2017) |
|  | RIF | (Hou et al. 2022) |
| impairing mitochondrial function | CPZ | (Hendriks et al. 2016) |
|  | KC | (Haegler et al. 2017) |
| cell death | CSA | (Nsengimana et al. 2022) |

| **Supplementary Table 3**. Summary of datasets used in model training. | | | |
| --- | --- | --- | --- |
| Dataset | Positive | Negative | Total number |
| Section A: DEG set | | | |
| Training | 75 | 76 | 151 |
| Testing | 33 | 32 | 65 |
| Section B: delta DEG set | | | |
| Training | 50 | 50 | 100 |
| Testing | 22 | 22 | 44 |
| Section A: shows the learning instances generated using the rawExpression-derived DEG-subsetted training data. Section B demonstrates learning examples obtained using the deltaChange-derived DEG-subsetted training data. | | | |

| **Supplementary Table 4.** KEGG pathways enriched by the time point-specific DEGs and deltaDEGs. | | | | | |  |
| --- | --- | --- | --- | --- | --- | --- |
| ID | Pathway description | p-value | ID | Pathway description | p-value | |
| Section A: DEG set | | | | | | |
| 8 hours | | | 8 hours | | | |
| hsa04110 | Cell cycle | 0.002 | hsa04060 | Cytokine-cytokine receptor interaction | 0.025 | |
| hsa04061 | Viral protein interaction with cytokine and cytokine receptor | 0.003 | hsa00010 | Glycolysis / Gluconeogenesis | 0.025 | |
| hsa05323 | Rheumatoid arthritis | 0.003 | hsa05417 | Lipid and atherosclerosis | 0.027 | |
| hsa05146 | Amoebiasis | 0.006 | hsa00220 | Arginine biosynthesis | 0.028 | |
| hsa00982 | Drug metabolism - cytochrome P450 | 0.008 | hsa04657 | IL-17 signaling pathway | 0.028 | |
| hsa00980 | Metabolism of xenobiotics by cytochrome P450 | 0.011 | hsa01230 | Biosynthesis of amino acids | 0.044 | |
| hsa00430 | Taurine and hypotaurine metabolism | 0.013 | hsa05204 | Chemical carcinogenesis - DNA adducts | 0.048 | |
| hsa00380 | Tryptophan metabolism | 0.015 | 24 hours | | | |
| hsa04668 | TNF signaling pathway | 0.016 | hsa04141 | Protein processing in endoplasmic reticulum | 0.000 | |
| hsa03320 | PPAR signaling pathway | 0.017 | hsa05164 | Influenza A | 0.006 | |
| hsa00620 | Pyruvate metabolism | 0.017 | hsa04213 | Longevity regulating pathway - multiple species | 0.035 | |
| hsa04146 | Peroxisome | 0.021 | hsa05160 | Hepatitis C | 0.042 | |
| hsa04936 | Alcoholic liver disease | 0.022 | hsa04115 | *p53 signaling pathway* | *0.061* | |
| hsa05134 | Legionellosis | 0.023 | hsa04976 | *Bile secretion* | *0.065* | |
| Section B: deltaDEG set | | | | | | |
| 8 hours | | | 24 hours | | | |
| hsa00900 | Terpenoid backbone biosynthesis | 0.004 | hsa00860 | Porphyrin metabolism | 0.009 | |
| hsa05134 | Legionellosis | 0.025 | hsa01240 | Biosynthesis of cofactors | 0.014 | |
| hsa04068 | FoxO signaling pathway | 0.027 | hsa00020 | Citrate cycle (TCA cycle) | 0.025 | |
| hsa01230 | Biosynthesis of amino acids | 0.047 | hsa04931 | Insulin resistance | 0.028 | |
| 24 hours | | | hsa05146 | Amoebiasis | 0.032 | |
| hsa01230 | Biosynthesis of amino acids | 0.000 | hsa03022 | Basal transcription factors | 0.035 | |
| hsa00260 | Glycine, serine and threonine metabolism | 0.001 | hsa00970 | Aminoacyl-tRNA biosynthesis | 0.039 | |
| hsa01200 | Carbon metabolism | 0.002 | hsa00670 | One carbon pool by folate | 0.039 | |
| hsa03320 | PPAR signaling pathway | 0.004 | hsa00120 | *Primary bile acid biosynthesis* | *0.069* | |
| Section A and B demonstrate the pathways that are significantly enriched in the time point-specific DEG and deltaDEG lists, respectively. *Italics* indicate marginally significant results (p-value<0.10). | | | | | |  |

| **Supplementary table 5**. Comparison of performance of models with the PFI-selected features using 100-round 5-fold cross-validation. | | | | | | | | | |
| --- | --- | --- | --- | --- | --- | --- | --- | --- | --- |
| Model | No. of PFI-selected features | Accuracy | AUC | Sensitivity | Specificity | PPV | NPV | F1-score |  |
| Section A: DEG set | | | | | | | | | |
| ADA | 9 | 0.869±0.016 | 0.934±0.01 | 0.861±0.023 | 0.879±0.024 | 0.879±0.021 | 0.865±0.021 | 0.866±0.016 |  |
| **KNN** | 92 | 0.964±0.01 | 0.964±0.01 | 0.955±0.014 | 0.973±0.014 | 0.973±0.015 | 0.956±0.014 | 0.963±0.011 |  |
| **XGBoost** | 18 | 0.922±0.013 | 0.972±0.005 | 0.917±0.022 | 0.929±0.014 | 0.929±0.014 | 0.919±0.02 | 0.921±0.014 |  |
| RF | 15 | 0.893±0.012 | 0.962±0.006 | 0.865±0.018 | 0.923±0.015 | 0.919±0.015 | 0.874±0.014 | 0.888±0.013 |  |
| **GAUSSIAN** | 39 | 0.956±0.008 | 0.992±0.002 | 0.959±0.01 | 0.954±0.011 | 0.954±0.011 | 0.958±0.01 | 0.955±0.009 |  |
| NNMLPC | 109 | 0.975±0.01 | 0.995±0.002 | 0.98±0.014 | 0.97±0.013 | 0.97±0.014 | 0.98±0.014 | 0.974±0.011 |  |
| Section B: delta DEG set | | | | | | | | | |
| **LR** | 17 | 0.954±0.009 | 0.987±0.003 | 0.935±0.011 | 0.977±0.012 | 0.974±0.014 | 0.937±0.011 | 0.952±0.01 |  |
| **SVMlinear** | 76 | 0.974±0.01 | 0.999±0.001 | 0.962±0.016 | 0.988±0.01 | 0.986±0.011 | 0.962±0.015 | 0.973±0.011 |  |
| **NNMLPC** | 15 | 0.895±0.015 | 0.97±0.009 | 0.893±0.022 | 0.899±0.028 | 0.9±0.025 | 0.895±0.017 | 0.892±0.016 |  |
| Results are expressed as mean ± standard deviation. **Bold models** were selected to extract the least number of features using the wrapper feature selection methods. AUC: area under the curve, PPV: positive predictive value and NPV: negative predictive value. Section A: results from the DEG set; Section B: outcomes from the deltaDEG set. | | | | | | | | | |

| **Supplementary Table 6.** Comparison of performance of models fed with wrapper method-selected features using 100-round 5-fold cross-validation. | | | | | | | | | | |
| --- | --- | --- | --- | --- | --- | --- | --- | --- | --- | --- |
| Model | Wrapper method | No. features | Accuracy | AUC | Sensitivity | Specificity | PPV | NPV | F1-score |  |
| Section A: DEG set | | | | | | | | | | |
| KNN | SFS | 24 | 0.961±0.009 | 0.961±0.008 | 0.957±0.013 | 0.965±0.011 | 0.964±0.011 | 0.958±0.013 | 0.96±0.009 |  |
|  | SBS | 23 | 0.95±0.01 | 0.95±0.01 | 0.949±0.011 | 0.943±0.016 | 0.959±0.015 | 0.941±0.017 | 0.96±0.014 |  |
|  | RFE | 25 | 0.936±0.012 | 0.937±0.012 | 0.924±0.019 | 0.95±0.018 | 0.948±0.018 | 0.926±0.017 | 0.934±0.013 |  |
| XGBoost | SFS | 14 | 0.919±0.015 | 0.965±0.006 | 0.92±0.022 | 0.919±0.018 | 0.92±0.017 | 0.921±0.019 | 0.918±0.016 |  |
|  | SBS | 11 | 0.911±0.012 | 0.965±0.007 | 0.911±0.012 | 0.919±0.014 | 0.906±0.018 | 0.92±0.015 | 0.904±0.019 |  |
|  | RFE | 15 | 0.921±0.014 | 0.974±0.005 | 0.92±0.022 | 0.924±0.016 | 0.925±0.015 | 0.921±0.02 | 0.92±0.015 |  |
| **GAUSSIAN** | SFS | 30 | 0.956±0.009 | 0.991±0.003 | 0.956±0.012 | 0.956±0.012 | 0.956±0.011 | 0.956±0.011 | 0.955±0.009 |  |
|  | **SBS** | **17** | 0.954±0.011 | 0.99±0.003 | 0.953±0.011 | 0.951±0.019 | 0.958±0.009 | 0.951±0.019 | 0.959±0.01 |  |
|  | RFE | 19 | 0.939±0.012 | 0.989±0.004 | 0.96±0.017 | 0.917±0.017 | 0.922±0.015 | 0.959±0.017 | 0.939±0.012 |  |
| Section B: deltaDEG set | | | | | | | | | | |
| SVMlinear | SFS | 33 | 0.968±0.006 | 0.995±0.002 | 0.971±0.007 | 0.966±0.009 | 0.964±0.012 | 0.97±0.008 | 0.966±0.008 |  |
|  | SBS | 21 | 0.977±0.01 | 0.993±0.002 | 0.961±0.016 | 0.995±0.007 | 0.993±0.009 | 0.96±0.018 | 0.976±0.01 |  |
|  | RFE | 62 | 0.983±0.008 | 1±0.001 | 0.973±0.013 | 0.995±0.007 | 0.993±0.009 | 0.972±0.013 | 0.982±0.009 |  |
| NNMLPC | SFS | 13 | 0.906±0.017 | 0.977±0.007 | 0.903±0.023 | 0.91±0.022 | 0.911±0.021 | 0.905±0.022 | 0.903±0.017 |  |
|  | SBS | 14 | 0.905±0.015 | 0.973±0.008 | 0.896±0.021 | 0.914±0.023 | 0.913±0.022 | 0.9±0.017 | 0.901±0.016 |  |
|  | RFE | 14 | 0.903±0.016 | 0.972±0.008 | 0.9±0.023 | 0.909±0.028 | 0.908±0.025 | 0.902±0.019 | 0.9±0.017 |  |
| **LR** | SFS | 13 | 0.954±0.01 | 0.986±0.003 | 0.932±0.014 | 0.979±0.01 | 0.976±0.014 | 0.934±0.013 | 0.951±0.012 |  |
|  | **SBS** | **13** | 0.958±0.01 | 0.984±0.004 | 0.941±0.015 | 0.978±0.009 | 0.975±0.012 | 0.942±0.015 | 0.956±0.011 |  |
|  | RFE | 14 | 0.964±0.01 | 0.987±0.004 | 0.945±0.013 | 0.985±0.011 | 0.982±0.014 | 0.947±0.014 | 0.962±0.012 |  |
| Results are expressed as mean ± standard deviation. **Bold models** and the least number of features were used for the external validation. AUC: area under the curve, PPV: positive predictive value and NPV: negative predictive value. Section A: results from the DEG set; Section B: outcomes from the deltaDEG set. | | | | | | | | | | |

| **Supplementary table 7**. General functions of genes identified by Gaussian model. | | |
| --- | --- | --- |
| Gene symbol | Gene name | General function |
| *ABHD15* | Abhydrolase Domain Containing 15 | Apoptosis* |
| *AMIGO2* | Adhesion Molecule With Ig Like Domain 2 | Apoptosis* |
| *ATP8B1* | ATPase Phospholipid Transporting 8B1 | PFIC1 and BRIC1 |
| *BLNK* | B Cell Linker | Apoptosis* |
| *BTG1* | BTG Anti-Proliferation Factor 1 | Apoptosis* |
| *CRP* | C-Reactive Protein | Inflammation* |
| *CTHRC1* | CollagenTripleHelixRepeatContaining1 | Apoptosis* |
| *CYP1A1* | Cytochrome P450 Family 1 Subfamily A Member 1 | BA composition* |
| *EIF2AK3* | Eukaryotic Translation Initiation Factor 2 Alpha Kinase 3 | ER stress* |
| *FABP1* | Fatty Acid Binding Protein 1 | BA release* |
| *GFPT1* | Glutamine--Fructose-6-Phosphate Transaminase 1 | ER stress*, Inflammation* |
| *MAP3K8* | Mitogen-Activated Protein Kinase Kinase Kinase 8 | Inflammation* |
| *OXTR* | Oxytocin Receptor | Apoptosis*, ICP |
| *RTP3* | Receptor Transporter Protein 3 | Liver cancer, chemical-induced liver injury |
| *TGM2* | Transglutaminase 2 | Apoptosis* |
| *TUBE1* | Tubulin Epsilon 1 | Liver cancer |
| *WIPI1* | WD Repeat Domain, Phosphoinositide Interacting 1 | Mitochondria impairment*, Autophagy* |
| BA: bile acid, ER: endoplasmic reticulum, ICP: intrahepatic cholestasis of pregnancy, PFIC1: progressive familial intrahepatic cholestasis type 1, BRIC1: benign recurrent intrahepatic cholestasis type 1 (BRIC1). *: chemical-induced cholestasis adverse outcome pathway network-related key event. | | |

**Included references in Supplementary Table 2.**

Amor D, Goutal S, Marie S, et al. (2018) Impact of rifampicin-inhibitable transport on the liver distribution and tissue kinetics of erlotinib assessed with PET imaging in rats. EJNMMI Res 8(1):81 <https://doi:10.1186/s13550-018-0434-0>

Chatterjee S, Richert L, Augustijns P, Annaert P (2014) Hepatocyte-based in vitro model for assessment of drug-induced cholestasis. Toxicol Appl Pharmacol 274(1):124-36 <https://doi:10.1016/j.taap.2013.10.032>

Dawson PA (2011) Role of the intestinal bile acid transporters in bile acid and drug disposition. Handb Exp Pharmacol(201):169-203 <https://doi:10.1007/978-3-642-14541-4_4>

Guo YX, Xu XF, Zhang QZ, et al. (2015) The inhibition of hepatic bile acids transporters Ntcp and Bsep is involved in the pathogenesis of isoniazid/rifampicin-induced hepatotoxicity. Toxicol Mech Methods 25(5):382-7 <https://doi:10.3109/15376516.2015.1033074>

Haegler P, Joerin L, Krahenbuhl S, Bouitbir J (2017) Hepatocellular Toxicity of Imidazole and Triazole Antimycotic Agents. Toxicol Sci 157(1):183-195 <https://doi:10.1093/toxsci/kfx029>

He K, Cai L, Shi Q, Liu H, Woolf TF (2015) Inhibition of MDR3 Activity in Human Hepatocytes by Drugs Associated with Liver Injury. Chem Res Toxicol 28(10):1987-90 <https://doi:10.1021/acs.chemrestox.5b00201>

Hendriks DF, Fredriksson Puigvert L, Messner S, Mortiz W, Ingelman-Sundberg M (2016) Hepatic 3D spheroid models for the detection and study of compounds with cholestatic liability. Sci Rep 6:35434 <https://doi:10.1038/srep35434>

Horikawa M, Kato Y, Tyson CA, Sugiyama Y (2003) Potential cholestatic activity of various therapeutic agents assessed by bile canalicular membrane vesicles isolated from rats and humans. Drug Metab Pharmacokinet 18(1):16-22 <https://doi:10.2133/dmpk.18.16>

Hou W, Nsengimana B, Yan C, Nashan B, Han S (2022) Involvement of endoplasmic reticulum stress in rifampicin-induced liver injury. Front Pharmacol 13:1022809 <https://doi:10.3389/fphar.2022.1022809>

Jazaeri F, Sheibani M, Nezamoleslami S, Moezi L, Dehpour AR (2021) Current Models for Predicting Drug-induced Cholestasis: The Role of Hepatobiliary Transport System. Iran J Pharm Res 20(2):1-21 <https://doi:10.22037/ijpr.2020.113362.14254>

Jemnitz K, Veres Z, Vereczkey L (2010) Contribution of high basolateral bile salt efflux to the lack of hepatotoxicity in rat in response to drugs inducing cholestasis in human. Toxicol Sci 115(1):80-8 <https://doi:10.1093/toxsci/kfq044>

Kock K, Ferslew BC, Netterberg I, et al. (2014) Risk factors for development of cholestatic drug-induced liver injury: inhibition of hepatic basolateral bile acid transporters multidrug resistance-associated proteins 3 and 4. Drug Metab Dispos 42(4):665-74 <https://doi:10.1124/dmd.113.054304>

Kolaric TO, Nincevic V, Smolic R, Smolic M, Wu GY (2019) Mechanisms of Hepatic Cholestatic Drug Injury. J Clin Transl Hepatol 7(1):86-92 <https://doi:10.14218/JCTH.2018.00042>

Kostrubsky VE, Strom SC, Hanson J, et al. (2003) Evaluation of hepatotoxic potential of drugs by inhibition of bile-acid transport in cultured primary human hepatocytes and intact rats. Toxicol Sci 76(1):220-8 <https://doi:10.1093/toxsci/kfg217>

Lau YY, Huang Y, Frassetto L, Benet LZ (2007) effect of OATP1B transporter inhibition on the pharmacokinetics of atorvastatin in healthy volunteers. Clin Pharmacol Ther 81(2):194-204 <https://doi:10.1038/sj.clpt.6100038>

Mita S, Suzuki H, Akita H, et al. (2006) Inhibition of bile acid transport across Na+/taurocholate cotransporting polypeptide (SLC10A1) and bile salt export pump (ABCB 11)-coexpressing LLC-PK1 cells by cholestasis-inducing drugs. Drug Metab Dispos 34(9):1575-81 <https://doi:10.1124/dmd.105.008748>

Morgan RE, Trauner M, van Staden CJ, et al. (2010) Interference with bile salt export pump function is a susceptibility factor for human liver injury in drug development. Toxicol Sci 118(2):485-500 <https://doi:10.1093/toxsci/kfq269>

Ni X, Gao Y, Wu Z, et al. (2016) Functional human induced hepatocytes (hiHeps) with bile acid synthesis and transport capacities: A novel in vitro cholestatic model. Sci Rep 6:38694 <https://doi:10.1038/srep38694>

Nsengimana B, Okpara ES, Hou W, Yan C, Han S (2022) Involvement of oxidative species in cyclosporine-mediated cholestasis. Front Pharmacol 13:1004844 <https://doi:10.3389/fphar.2022.1004844>

Sharanek A, Azzi PB, Al-Attrache H, et al. (2014) Different dose-dependent mechanisms are involved in early cyclosporine a-induced cholestatic effects in hepaRG cells. Toxicol Sci 141(1):244-53 <https://doi:10.1093/toxsci/kfu122>

Sharanek A, Burban A, Burbank M, et al. (2016) Rho-kinase/myosin light chain kinase pathway plays a key role in the impairment of bile canaliculi dynamics induced by cholestatic drugs. Sci Rep 6:24709 <https://doi:10.1038/srep24709>

Tolosa L, Pinto S, Donato MT, et al. (2012) Development of a multiparametric cell-based protocol to screen and classify the hepatotoxicity potential of drugs. Toxicol Sci 127(1):187-98 <https://doi:10.1093/toxsci/kfs083>

Vatakuti S, Olinga P, Pennings JLA, Groothuis GMM (2017) Validation of precision-cut liver slices to study drug-induced cholestasis: a transcriptomics approach. Arch Toxicol 91(3):1401-1412 <https://doi:10.1007/s00204-016-1778-8>

Wu Q, Duan Z, Huang L, Li Z (2022) Kuhuang injection exerts a protective effect by activating PPAR-gamma in an in vitro model of chlorpromazine-induced cholestatic liver injury constructed by tissue engineering. Pharm Biol 60(1):1679-1689 <https://doi:10.1080/13880209.2022.2110128>

Xu BY, Tang XD, Chen J, Wu HB, Chen WS, Chen L (2020) Rifampicin induces clathrin-dependent endocytosis and ubiquitin-proteasome degradation of MRP2 via oxidative stress-activated PKC-ERK/JNK/p38 and PI3K signaling pathways in HepG2 cells. Acta Pharmacol Sin 41(1):56-64 <https://doi:10.1038/s41401-019-0266-0>

Yew WW, Chang KC, Chan DP (2018) Oxidative Stress and First-Line Antituberculosis Drug-Induced Hepatotoxicity. Antimicrob Agents Chemother 62(8) <https://doi:10.1128/AAC.02637-17>

Zhang J, He K, Cai L, et al. (2016) Inhibition of bile salt transport by drugs associated with liver injury in primary hepatocytes from human, monkey, dog, rat, and mouse. Chem Biol Interact 255:45-54 <https://doi:10.1016/j.cbi.2016.03.019>
